# How journalists and experts metaphorically frame emerging information technologies: The case of cyberinfrastructure for big data

**DOI:** 10.1177/0963662520952542

**Published:** 2020-08-30

**Authors:** Ellen Droog, Christian Burgers, Kerk F. Kee

**Affiliations:** Vrije Universiteit Amsterdam, The Netherlands; Vrije Universiteit Amsterdam, The Netherlands; University of Amsterdam, The Netherlands; Texas Tech University, USA

**Keywords:** cyberinfrastructure, emerging information technology, frame-building, framing, journalists, metaphors, science communication, technology experts

## Abstract

Journalists and experts contribute to the creation of frames (frame-building) of innovations. However, little is known about the specific contribution of these different societal actors to the frame-building of emerging information technologies. This article focuses on a specific emerging information technology – cyberinfrastructure for big data. In particular, we investigated the role of metaphors in the frame-building of cyberinfrastructure during its early development, and contrast the metaphorical framing of cyberinfrastructure by journalists in a corpus of US news texts (Study 1) with the metaphorical framing of experts in a corpus of interviews (Study 2). Results show considerable differences between the frames by journalists and experts in the frame-building process. Journalists, to a great extent, employ their own frames in conceptualizing cyberinfrastructure rather than drawing on the frames used by experts. Future research should investigate the impact of these different metaphorical frames on audience members.

## 1. Introduction

To make sense of abstract and complex technologies, people often use metaphorical frames (Burgers, 2016). Comparing a new technology with something others are already familiar with makes the technology easier to comprehend. Metaphorical frames are widely used in people’s conceptualization of the Internet, which is, for instance, metaphorically referred to as a physical tool (Markham, 2003), a highway (Palmquist, 2001) or a three-dimensional physical space that you can enter and leave (Matlock et al., 2014). Metaphorical framing of established technologies such as the Internet has received a lot of attention (Markham, 2003; Palmquist, 2001), while little is known about how people metaphorically talk about emerging information technologies during their early development.

The majority of metaphor studies within the context of information technologies were conducted when the technologies had already been completely developed and implemented (Matlock et al., 2014; Puschmann and Burgess, 2014). Therefore, at the time of being studied, metaphorical frames had already been formed, and were widely known and used. Emerging technologies, in contrast, do not yet have established frames (Burgers, 2016). Emerging information technologies are therefore suitable for studying frame-building (how new frames are created) and sense-making of innovations (Burgers, 2016; Scheufele, 1999). The novel perspective of this study is that we investigate frame-building of a technology that is still in its early phase of development and not yet fully known by the general public at the time of research.

Moreover, studying how different societal actors use metaphorical frames within the context of an emerging technology can provide insight into the frame-building process. However, most studies examined metaphor use by lay users of the technology, and not by experts involved in the development of that technology (Matlock et al., 2014; Palmquist, 2001). The aim of this study is to investigate the (dis)similarities in the metaphorical framing of emerging information technologies between journalists and experts. In this study, we investigate whether the frames used by technology experts to explain an emerging information technology also make their way into media coverage of that technology. Thus, we contrast the metaphorical frames used by journalists to conceptualize the emerging information technology of cyberinfrastructure with those used by technology experts.

## 2. Theoretical framework

### Metaphorical framing of emerging information technologies and innovations

Metaphors are one of the most important communicative tools for talking about complex topics such as new technologies (Bauer and Bogner, 2020; Jaspal and Nerlich, 2014; Luokkanen et al., 2014), science (Knudsen, 2005; Leydesdorff and Hellsten, 2005) or societal issues such as climate change (Woods et al., 2012). Metaphors are defined as ‘cross-domain mappings’ (Lakoff and Johnson, 2003 [1980]), in which information from a source domain is mapped onto a target domain. For instance, the metaphor ‘the Internet is a highway’ (Palmquist, 2001) maps elements from the source domain of ‘highway’ onto the target domain of ‘the Internet’. Conceptual Metaphor Theory (CMT; Lakoff and Johnson, 2003 [1980]) proposes that linguistic metaphors are not used randomly, but cluster in larger conceptual structures (conceptual metaphors). For instance, the highway metaphor may be part of a conceptual metaphor for talking about digital spaces in terms of physical spaces, which is also reflected in expressions such as ‘I went to this homepage’ or ‘I am surfing the Web’ (Matlock et al., 2014). In this way, metaphors provide frames of thinking about innovations (Lakoff and Johnson, 2003 [1980]), and, by reducing the complexity, help people conceptualize and talk about these new technologies.

A specific technology can be conceptualized through different (and sometimes competing) metaphors. The Internet is, for example, often described as a ‘highway’ (Palmquist, 2001), a ‘tool’ (Markham, 2003) or a ‘frontier’ (Markham, 2003). Other technologies such as the first iPhone, were labelled in religious terms as the ‘Jesus phone’, or ‘the holy grail of all gadgets’ (Campbell and La Pastina, 2010). How people talk about a technology in its early phase of development can have a large influence on how people see and use that technology subsequently (Markham, 2003). However, many studies have only investigated the metaphors used to conceptualize established information technologies (Matlock et al., 2014; Puschmann and Burgess, 2014), while little attention has been given to what kind of metaphors are used to talk about emerging information technologies. In the current article, we focus on the metaphorical framing of an information technology that is still under early development.

The question of how people conceptualize emerging information technologies relates to frame-building (Scheufele, 1999). Frame-building refers to the process through which societal actors create or change frames, and the factors that can shape this process (Brüggemann, 2014; Scheufele, 1999). Studies on frame-building focus on why and when societal actors decide to use which frames for what purpose (Scheufele, 1999). Generally, a frame is defined as the mechanism for ‘select[ing] some aspects of a perceived reality and mak[ing] them more salient in a communicating text, in such a way as to promote a particular problem definition, causal interpretation, moral evaluation, and/or treatment recommendation for the item described’ (Entman, 1993: 53). Scholars generally argue that a frame consists of two elements – a framing device (linguistic packaging of the frame) and a reasoning device (the frame’s conceptual content; Joris et al., 2014). Metaphorical framing is based on the idea that metaphors can work as both a framing and reasoning device (Burgers et al., 2016). Metaphorical frames are used more often when discussing new and complex topics rather than already established ones (Burgers et al., 2016).

One group of important societal actors is journalists. Journalists play an important role in creating metaphorical frames for the general public, by functioning as gatekeepers for specific frames. Moreover, other societal actors such as developers and experts of a technology can influence the frame-building process as well. The metaphorical frames are partially shaped by the different backgrounds of the social actors involved (Scheufele, 1999). Social actors can hold different perspectives about various aspects of technology due to their different roles, general knowledge of the technology, and world views (Kee, 2015; Wear, 1999). Hence, studying how different societal actors use metaphorical frames within the context of an emerging information technology can provide insight into how new frames are built and how different societal actors contribute to this process.

News frames are constructed through a negotiation of meaning between journalists and other societal actors (Cook, 1998; Gans, 1979). Journalistic framing practices can be placed on a continuum between frame-sending (passively passing on frames provided by other societal actors) and frame-setting (passing on frames solely based on journalists’ own interpretations; Brüggemann, 2014). The actions of most journalists are somewhere on the middle of this continuum, because, to some degree, journalists use frames of other societal actors, but simultaneously rely on their own frames in creating news (Brüggemann, 2014). Generally, journalists observe science, such as technological innovations, according to a set of rules that may be different than the rules of the ‘social system’ (i.e. the technology experts) being observed (Kohring, 2005). According to Brüggemann (2014), however, research on frame-building is underdeveloped. Thus, a major challenge for scholars is to identify conditions that determine the degree of journalistic frame-setting practices (Brüggemann, 2014). By contrasting journalists’ metaphorical framing of an emerging information technology with those of experts, we investigate how these different societal actors contribute to the creation of frames, and whether journalists invoke certain experts’ metaphorical frames, or if they mainly use their own frames when creating news about emerging information technologies. This can also show what kind of metaphors would foster a shared understanding of the form and function of the technology between experts and journalists.

The use of metaphorical frames does not entail a one-size-fits-all approach. Studies indicate that domain experts and journalists (often novices to the specific new technology) differ in their metaphor use. Academics (domain experts) generally use fewer metaphors than journalists (Skorczynska and Deignan, 2006; Steen et al., 2010). Moreover, Skorczynska and Deignan (2006) showed that scientific articles about economics contained a narrower range of metaphors than popular articles about economics, and that there is also little overlap in metaphors between these discourses. Metaphors used in academic articles tended to be more genre-specific. Journalistic metaphors, by contrast, tended to be more general in language as a whole (metaphors that are not bound to a specific subject and are used in everyday language). By studying the metaphor use of technology professionals (experts) and technology novices (journalists), we can investigate how different societal actors make sense of an emerging information technology. This provides a better understanding of how these different societal actors influence the process of frame-building, and if and how journalists invoke experts’ metaphorical frames.

Furthermore, most metaphors in ordinary language use are conventional (Lakoff and Johnson, 2003 [1980]; Steen et al., 2010). These are metaphors that are so frequently used that their metaphorical meaning is included in the dictionary (Steen et al., 2010), such as the metaphorical meaning of ‘surf’ in expressions like ‘I am surfing the Web’. By contrast, novel metaphors involve a new cross-domain mapping, such as the word ‘cake’ in the metaphorical expression ‘the internet is like a high-tech cake made up of layers of wires, software and protocols that transmit information’ (Lacy, 2018). Research on the novelty of metaphors shows that expert writers use more novel metaphors compared to novice writers, and that more novel metaphors are constructed when individuals describe their own feelings rather than those of others (Williams-Whitney et al., 1992). Compared to conventional metaphors, the use of novel metaphors can have a positive influence on persuasiveness (Sopory and Dillard, 2002) and can stir critical thinking (Hansen et al., 2011). In the current article, we focus on whether emergent information technologies are conveyed through novel or conventional metaphors.

Besides this distinction, metaphors can also be contrasted based on their level of abstraction (McCabe, 1988). According to Iliev and Axelrod (2017), abstraction consists of two dimensions: concreteness and precision. Concreteness can be referred to as physicality. Thus, physical entities (e.g. ‘pen’) which can be experienced through the senses, are more concrete than non-physical entities (e.g. ‘feelings’). Precision is defined as specificity, which means that the more information is provided about the entity, the more precise it is (Iliev and Axelrod, 2017). In this perspective, ‘fountain pen’ is more specific than ‘pen’. When concrete metaphors are used in a new ICT application, people have a higher usage intention, a higher satisfaction and a higher preference for that application compared to an application with abstract metaphors (Zhou et al., 2017). However, it is also not known how concrete and precise the metaphors used to conceptualize an emerging information technology are. In the current article, we focus on how emergent information technologies are conveyed through such abstract and specific metaphors. By studying the different characteristics of metaphors that are used to talk about an emerging information technology, we can investigate how they are used in the sense-making of such complex technologies, and whether different types of societal actors might differ in their use of these different types of metaphors.

### Metaphorical framing of cyberinfrastructure

Our case study focuses on cyberinfrastructure, an emerging, complex and evolving information technology (Kee et al., 2011), also known as the ‘next-generation Internet’ (Foster et al., 2001). Due to improvements in computing and information technologies, researchers are able to answer new and more complex scientific research questions (Cyberinfrastructure Council, 2007) and to develop new ways to collaborate on research projects through faster, better and larger scientific research and computational capabilities on big data (Hey and Trefethen, 2005). Cyberinfrastructure is the platform for this large-scale science (Kee et al., 2011) and is composed of a network of supercomputers, big data, visualization of data, computational simulation, interdisciplinary scientists and multiple institutions (e.g. universities and industries). This enables virtual environments and virtual organizations (VOs) to conduct data-intensive and large-scale science (Atkins, 2003; Kee, 2017; Kee et al., 2011). In such VOs, geographically dispersed scientists (as lead users) and technologists (as developers) from various disciplines can collaborate on projects across institutions and time zones by sharing data, expertise and ideas without needing to work face-to-face (Atkins, 2003). In these projects, people create tools and programs that can advance science with big data, by for example, analysing the structure of any protein, simulate extreme weather conditions such as tornados or predict climate change by calculating ocean temperatures in a few years (Cyberinfrastructure Council, 2007).

Cyberinfrastructure is expanding the traditional use of the Internet. While the Internet was first created for communication and information sharing between different military personnel, scientists and universities (‘ARPANET’, Kahn et al., 1997), cyberinfrastructure’s purpose lies in the next level of communication and information sharing between scientists and universities, as cyberinfrastructure connects multiple supercomputers into a coherent consortium for big data (Kee et al., 2011). It is expected that cyberinfrastructure will also be adopted in the future for commercial, public and private use (Kee et al., 2011), which puts an even greater emphasis on the need to create a shared understanding of the form and function of this emerging technology.

By contrasting journalists’ metaphorical framing of cyberinfrastructure with the metaphorical framing of experts, we investigate how these different stakeholders make sense of an emerging information technology, and what and how different societal actors contribute to the creation of frames (Scheufele, 1999). It can also reveal whether frame creation arises through an interaction between journalists and other societal actors (Brüggemann, 2014; Cook, 1998; Gans, 1979), or whether journalists primarily draw on their own frames when creating news about emerging technologies. Studying experts’ metaphorical framing also provides an opportunity to study the metaphor use of technology experts (i.e. technologists and lead users in the case of cyberinfrastructure) as the pioneers involved in this emerging and evolving technology, instead of subsequent users when the technology is mature and established (Matlock et al., 2014).

We conducted two studies to investigate journalists’ and experts’ metaphorical framing of cyberinfrastructure. Study 1 focused on the metaphorical framing of journalists using a corpus of US news text on cyberinfrastructure, while Study 2 focused on the metaphorical framing of experts in a corpus of interviews with stakeholders involved in the development of cyberinfrastructure. Together, these two studies addressed the following research question: *What are the (dis)similarities in the metaphorical framing of cyberinfrastructure between journalists and technology experts?*

## 3. Study 1: Metaphorical frames used by journalists

### Method

#### Materials

The LexisNexis database was used to retrieve American news articles about cyberinfrastructure. The keyword ‘cyberinfrastructure’ was used to search the database for articles from newspapers published throughout the country. This initial search identified more than 3000 articles; however, because LexisNexis cannot display more than 1000 articles at once, only the first 1000 articles (which LexisNexis sorted automatically based on relevance) were used for further steps of the analysis. The next step involved the removal of duplicates. Thereafter, the articles were manually screened by reading the headlines and by screening the text to ensure that the article was indeed focused on cyberinfrastructure. A total of 219 articles fulfilled these conditions. Due to the manual coding procedure of metaphorical words, 15 articles (2003–2016) were randomly^1^ chosen for further analysis (for an overview, see online Appendix A^2^). The total corpus of these 15 articles comprised 15,023 words.

#### Coding procedure

We analysed the data according to the methodology of critical metaphor analysis (Charteris-Black, 2004). This method consists of three different steps: identification, interpretation and explanation. The identification phase (Charteris-Black, 2004) consisted of manually identifying metaphor-related words (MRWs) within the news articles. This process was carried out by applying the Metaphor Identification Procedure of the Vrije Universiteit (MIPVU; Steen et al., 2010). The unit of analysis in the MIPVU is the lexical unit (LU). LUs are often the same as single words, with a small number of exceptions (for details, see Steen et al., 2010). The LUs that needed to be analysed as single units were collapsed into single cases, which resulted in a total of 14,654 LUs.

MRWs are words whose contextual meanings contrasts with their basic meanings and can be understood by making a comparison with it. The basic sense is defined as the most concrete (in that it can be touched, seen, heard, smelled or tasted), human-oriented and most precise (instead of vague) meaning of a word. The contextual meaning is the meaning of that LU within the specific setting in which it is used (see online Appendix B for more detailed information).

In the interpretation phase (Charteris-Black, 2004), MRWs were manually clustered into one of 21 general conceptual source domains (e.g. movement, architecture or technology) and their associated conceptual sub-domains based on their basic meaning. These domains were based on the UCREL Semantic Analysis System (USAS). This was done to determine what kind of conceptual source domains were recurrent in the language of journalists in explaining cyberinfrastructure. The explanation phase (Charteris-Black, 2004) consisted of identifying larger patterns (metaphorical frames) in the data and specifically entailed the categorization of different types of metaphors into subgroups (abstraction and conventionality/novelty).

#### Intercoder reliability

A second annotator analysed 20% of the data according to the same coding procedure (identification/interpretation phase; Charteris-Black, 2004). Reliability analysis was conducted to examine the extent of agreement between the two annotators. For the metaphor coding (identification phase), a Cohen’s kappa of .66 (indicating substantial agreement; Landis and Koch, 1977) was achieved. Online Appendix C shows that reliability differed considerably across conceptual domains; interpretation phase). Our qualitative analysis, therefore, mainly focused on the larger patterns in the data (i.e. the explanation phase; Charteris-Black, 2004) for the domains which received an acceptable degree of reliability.

### Results

#### Frequency of (conceptual) metaphors

We found that 3892 of 14,654 LUs (26.6%) were used metaphorically. More than one in every four words was a metaphor. This high percentage could reflect the complexity of cyberinfrastructure, in that metaphors are needed to reduce complexity for understanding. The analysis also shows that all 21 conceptual domains were used when framing cyberinfrastructure. A total of 69 conceptual sub-domains could be found in the analysis. This indicates that journalists use a wide variety of metaphors to talk about cyberinfrastructure. These domains and several linguistic examples are presented in online Appendix D. Online Appendix E includes the proportion of all linguistic metaphors that belong to a particular conceptual domain.

#### Conventional metaphorical frames

##### Business as usual

Generally, journalists conceptualized cyberinfrastructure with already existing and conventional (technological) metaphorical frames. However, they applied these existing frames in two different ways. Typically, journalists portrayed cyberinfrastructure as ‘business as usual’ by using frames about ‘known’ concepts, borrowed from other technologies such as the Internet and technological concepts such as big data:

1. ‘This requires a high-performance data **freeway** system in which we use optical **light paths** to connect data generators and users’. (SNS03082015)
2. ‘The NSF [National Science Foundation] can’t **develop** a national cyberinfrastructure in a vacuum’. (CW05052006)

Examples 1 and 2 contain conventional metaphors for technology. In Example 1, data generators are connected with users, similar to the ways the roads of a freeway are connected with different places, which shows the complex structures behind the technology. This is a frequently recurring metaphor in people’s conceptualization of the Internet, in which the Web is often compared to the highway (Palmquist, 2001). In Example 2, a conventional Internet metaphor is used by conceptualizing cyberinfrastructure as a physical object (e.g. a machine/house) that can be developed. This metaphor relates to the Internet because people also saw the information highway (the Internet) as something that could be built (Montreuil, 2011). These examples show that journalists typically did not create novel metaphors but exploited already conventional technological ones, to portray cyberinfrastructure as business as usual.

##### Disruption of current practices

Furthermore, journalists also used conventional technological metaphors to portray cyberinfrastructure as a disruption of current practices by using frames about ‘unknown’ or disruptive concepts:

3. ‘You have a **flood** of data that threatens to swamp our capacity to preserve, analyse, and apply it’. (TCHE05012007)
4. ‘i-Plant [a VO], taming the big data **beast**’. (SNS23072015)

Examples 3 and 4 also employ conventional technological frames. However, in these instances, cyberinfrastructure is portrayed as unknown or scary. Example 3 conceptualizes cyberinfrastructure as something unknown, but also as something scary by portraying data as a threat. Example 3 shows that similar to a tsunami that can swamp an area of land, large amounts of data can swamp our ability to deal with it. The same is true for Example 4, in that big data is conceptualized as an uncontrolled beast that needs to be tamed by people in the VO. This makes the use of cyberinfrastructure for big data sound frightening and disruptive, by suggesting that big data is unpredictable and almost out of human control. The Internet is sometimes also metaphorically described as an information beast (Senyuva and Kaya, 2013). These examples show how journalist used already conventional technological frames to portray cyberinfrastructure as a disruption of current practices.

#### Abstraction

In addition, a pattern of the abstractness of the metaphorical frames emerged. According to Iliev and Axelrod (2017), abstraction consists of two dimensions: concreteness (physicality) and precision (specificity). In relation to cyberinfrastructure, most metaphorical frames used by journalists were concrete but unprecise, as is represented in the following examples:

5. ‘These **tools** are not simply faster – they are also fundamentally superior’. (TCHE05012007)
6. ‘Researchers whose work requires powerful computing **resources**’. (SNS07082005)

Examples 5 and 6 both contain metaphors that are concrete (physical) but unprecise. In Example 5, the use of the metaphor ‘tool’ is concrete because a tool is a physical object of equipment. Similar to the ways in which tools like hammers or drills are designed to do a particular job (Markham, 2003), cyberinfrastructure is a tool that is designed to conduct scientific research with big data, leading to revolutionary discoveries. Talking about ‘resources’ in Example 6 is also concrete, because this metaphor reflects an existing entity. Most resources are physical in a solid, liquid or gas form. Similar to a machine which requires energy to operate (Puschmann and Burgess, 2014), this metaphor implies that, for scientists to do their work with big data properly, they need cyberinfrastructure as necessary resources.

However, the ‘tool’ metaphor in Example 5 is unprecise because it does not specify the type of tool. The metaphor provides less information than when it was explicitly stated if the tool was more like a hammer, a screw-driver or a drill. This is also true for Example 6, which does not explicitly state which aspects of cyberinfrastructure is the necessary ‘resource’. Overall, these examples show that it seemed difficult for journalists to generate precise metaphors for topics that are complex and difficult to understand. To investigate if the metaphorical framing of journalists differs from the metaphorical framing of experts, the next study reports on how many and what kind of metaphorical frames they use to conceptualize cyberinfrastructure.

## 4. Study 2: Metaphorical frames used by technology experts

### Method

#### Materials

The data for Study 2 constituted 147 interviews with 116 stakeholders, some of whom were interviewed twice. The stakeholders were based in the US/Europe and were involved in various roles in cyberinfrastructure development (e.g. technological developers, lead scientists or supercomputing centre administrators). The interviews were conducted originally for another study (Kee et al., 2016), but were re-analysed for Study 2 in this article. Interviews were conducted either via face-to-face or the telephone, and transcribed for research purposes. The total corpus of transcribed interviews comprised 902,997 words.

Interviews were conducted according to a pre-designed protocol. This protocol included three standard questions. Interviewees were asked to talk about (1) key activities during the development and use of computational tools in cyberinfrastructure-enabled VOs, (2) key people, objects and interactions in such VOs and (3) factors that impact cyberinfrastructure tool adoption and diffusion within the scientific community. The semi-structured nature of the interviews allowed interviewers flexibility to adapt to the flow of the interviews.

#### Coding procedure

We analysed the data according to the same critical metaphor analysis method (Charteris-Black, 2004) as in Study 1. However, because of the large corpus size of more than 900,000 words, the steps taken in the identification phase (Charteris-Black, 2004) were slightly different. We decided to primarily focus on direct metaphors. Direct metaphors often take the form of A (target) is like B (source), and are less prevalent compared to MRWs and are also somewhat easier to find (Steen et al., 2010). The search for direct metaphors was conducted in two different ways. First, interviews were examined with AntConc (Anthony, 2019), a software program able to search texts for specific words. In this case, the interviews were automatically searched for metaphor flags, words that can indicate the presence of a marked direct metaphor. Direct metaphors (compared to indirect metaphors) are defined by the presence of such a signal for comparison and because their source domains are directly expressed in the text (Steen et al., 2010). Examples of metaphor flags are ‘as (if)’, ‘like’, ‘metaphorical(ly)’, ‘literal(ly)’, ‘(so)called’, ‘some sort of’, ‘in a way’ or ‘kind of’.

All 147 interviews were searched for metaphor flags which resulted in 14,368 sentences containing one of these words. These sentences were manually read to determine if they contained the analogical form of a direct metaphor in the form of A is (like) B (e.g. cyberinfrastructure is like a platform). Thereafter, we determined if the analogical form was metaphorical or not. This was also done according to the steps of MIPVU (see Study 1; Steen et al., 2010).

In the interpretation phase (Charteris-Black, 2004), all direct metaphors were grouped into conceptual domains, based on grounded theory (Strauss and Corbin, 1990). These conceptual domains were created through an iterative process of reading and re-reading all the direct metaphors to identify conceptual domains. This was done to determine what kind of conceptual domains were recurrent in the language of experts in explaining cyberinfrastructure. The explanation phase (Charteris-Black, 2004) was similar to Study 1 and consisted of identifying larger patterns in the data and the categorization of different types of metaphors into subgroups based on abstraction and novelty.

#### Reliability

A second annotator analysed 10% of the 14,368 sentences containing a metaphor flag, according to the same coding procedure. Reliability analysis was conducted to examine the extent of agreement between the two annotators. For the direct metaphor coding, a Cohen’s kappa of .61 (indicating substantial agreement; Landis and Koch, 1977) was achieved.

### Results

#### Frequency of (conceptual) metaphors

A total of 14,368 sentences contained a metaphor flag. Of these 14,368 sentences, 931 sentences contained the analogical form of a direct metaphor. Moreover, 661 of those 931 analogical sentences were metaphorical in nature, and the other 270 analogical sentences were non-metaphorical. The amount of direct metaphors may seem like a small number compared to the first study. However, previous research indicates that only between 0.1% and 0.4% of all words used across registers (including academic registers) are direct metaphors in the form of A is (like) B (Steen et al., 2010).

Direct metaphors were grouped into 22 conceptual domains. These domains and several linguistic examples are presented in online Appendix F. Online Appendix G presents the proportion of all linguistic direct metaphors that belong to a particular conceptual domain. When contrasting the amount of conceptual domains used by experts with the 69 conceptual sub-domains used by journalists, the results indicated that experts use a more specific set of different kinds of metaphors than journalists.^3^

#### Novel yet familiar metaphorical frames

Generally, experts used novel metaphorical frames in describing cyberinfrastructure, but in doing so, also exploited familiar source domains. This implies that experts used metaphors that are new for describing cyberinfrastructure, but portrayed the technology as similar to ‘everyday life’ at the same time. However, readers would probably not automatically link (some of) these familiar source domains to technological concepts, as is reflected in the following examples:

7. ‘But I have like this **cookbook** or **recipe**, and that’s what I use to get to whatever the result is. I mean, the data can change and the results can change, but I have to make sure I document the process [with cyberinfrastructure] for myself’.
8. ‘Picking a piece of software [for cyberinfrastructure] is like **entering a relationship.** You really want to–this is a serious thing’.

Examples 7 and 8 show a new way of conceptualizing a technology, while using familiar source domains. In Example 7, carrying out research in a cyberinfrastructure is compared to using a cookbook or recipe to create a dish, which many people do every day. This indicates that cyberinfrastructure is easy to use because you can follow a standard recipe and achieve a good result (a delicious dish). Example 8 compares cyberinfrastructure with relationships. For most people, this is a new and different domain for conceptualizing a technology, yet it is a familiar source domain from everyday life. Picking a piece of software is quite a commitment and a large time investment, and the time that it takes to get to know someone intimately can be compared to the time you need to get to know how to use the tool. These examples show that experts generally created novel metaphorical frames when describing cyberinfrastructure, but exploited conventional source domains, which makes cyberinfrastructure appear familiar.

#### Abstraction

A recurrent pattern of the abstractness also emerged in this analysis. However, in contrast to the first study, most metaphorical frames used by experts were both concrete (physical) and precise (specific; Iliev and Axelrod, 2017), which is reflected in the following examples:

9. ‘**Tools** are often built for a specific purpose – like a **hammer’s use is generally to push in nails and it’s not meant to screw things in. When it comes to virtual [**cyberinfrastructure] tools, it’s kind of different, you want like your **Swiss Army knife** instead’.
10. ‘We want to find a drug that we want to bind to it. We will basically throw at it thousands of three-dimensional coordinates for small chemicals and the program [in cyberinfrastructure] will determine if the compounds fit on it. It is kind of like **a doorknob with a lock on it** like a protein target. We are basically testing thousands and thousands of different **keys** to see which one fits’.

Examples 9 and 10 both contain metaphors that are concrete (physical) and precise (provide a lot of information). Like in Example 5, the use of the metaphor ‘tool’ in Example 9 is concrete because a tool can be physically held. Similarly, in Example 10, the use of software made in a cyberinfrastructure is compared to a closed door of a house (physical building) with a doorknob with a lock on it, which is concrete, because doorknobs and locks are physical objects. This latter metaphor shows that by finding the right key you can unlock the door and enter the building, which means finding the right protein, and achieving groundbreaking results with cyberinfrastructure.

However, unlike Example 5, the use of the metaphor ‘tool’ in Example 9 is precise, because specific tools such as hammers and Swiss army knives are mentioned. This provides more information about the entity than the generic use of ‘tool’ in Example 5; experts want their virtual tools to be able to do many different things, similar to a Swiss army knife which has multiple functions. The same is true for Example 10, where the ‘doorknob’ metaphor is precise because it provides a large amount of information about the software, and a detailed description of how the software works. These examples show that experts tended to generate concrete and precise metaphors for this complex and emerging information technology.

## Conclusion and discussion

The goal of this study was to contrast journalists’ metaphorical framing of cyberinfrastructure with those of experts. The results of these studies have important implications, in that – to the best of our knowledge – this is the first study that provides an overview of the metaphorical frames with which cyberinfrastructure is conceptualized. The metaphors found in Study 1 were clustered into 21 general and 69 conceptual sub-domains. Our results suggest that, at the beginning of the frame-building process, journalists used a large variation of metaphorical source domains in their conceptualization of this emerging information technology. We expect that this variation will decrease over time as cyberinfrastructure becomes more familiar, because journalists will become better adapted to the technology, diminishing the need for many different metaphorical source domains (Matlock et al., 2014).

The metaphors found in Study 2 were clustered into 22 general conceptual domains. In contrast to the 69 conceptual sub-domains used by the journalists, experts use a more specific set of different metaphorical source domains than journalists at the beginning of the frame-building process. This is in line with previous research (Skorczynska and Deignan, 2006), which showed that domain experts use fewer types of metaphors than journalists. This gives us a first indication, that in the process of creating frames, journalists generate many source domains that are not used by experts, which means that they, to an extent, draw on their own frames when conceptualizing cyberinfrastructure.

The studies show that both journalists and experts portrayed cyberinfrastructure as something ‘known’, but in two different ways. Generally, to describe cyberinfrastructure, journalists used metaphorical frames that are conventional to describe established technologies, such as the Internet. These findings confirm earlier predictions by Markham (2003) who expected that future information technologies would be ‘put under the general umbrella category of the Internet’ (p. 2). In Markham’s (2003) view, any emerging information technology ‘can be seen as a new addition to the network of highways’ (p. 3), which is why cyberinfrastructure might be conceptualized as a freeway. An explanation for the frequent use of these Internet metaphors could be that cyberinfrastructure is also known as the next-generation Internet (Foster et al., 2001). Journalists may ground their understanding of cyberinfrastructure in their knowledge of the traditional Internet and, therefore, use the same kind of metaphorical frames to describe the emerging information technology. This result offers initial insights into the frame-building process (Brüggemann, 2014; Scheufele, 1999) for this emerging information technology. Journalists used already conventional metaphors from another information technology (the Internet) to describe cyberinfrastructure, suggesting that at the beginning of the frame-building process there are, so far, no metaphorical frames in the media that specifically belong to cyberinfrastructure yet.

According to Diffusion of Innovations Theory (DIT; Rogers, 2003), portraying a new technology as similar to existing technologies can be problematic for successful adoption. For any innovation to be adopted, DIT argues that it needs to have a relative advantage over an existing technology. If cyberinfrastructure is portrayed as similar to the Internet, potential users might not recognize any relative advantage, or the advantage is too abstract to understand, and thus hesitate on adoption. Therefore, from a diffusion perspective, this conceptualization might not be the best way to metaphorically frame cyberinfrastructure.

In contrast to journalists, experts used more novel metaphorical frames to conceptualize cyberinfrastructure, but they also made the information technology appear familiar by utilizing conventional source domains. DIT predicts that this can be helpful in the successful adoption of technologies in two ways. First, conceptualizing cyberinfrastructure with new metaphorical frames puts an emphasis on the unique aspects of cyberinfrastructure, making it easier to explicate the relative advantage of this technology. Second, DIT proposes that innovations that seem difficult to understand and use are typically poorly adopted. Comparing cyberinfrastructure to simple everyday experiences may give the impression that the technology is easy to understand and use, which reduces its perceived complexity (Rogers, 2003). This approach to conceptualize cyberinfrastructure may be a proper frame to stimulate adoption and diffusion. The fact that experts used novel technological metaphorical frames indicates that, at the beginning of the frame-building process, there are metaphorical frames that specifically belong to the emerging information technology of cyberinfrastructure. This result gives a second indication that, in creating frames, journalists primarily draw on their own metaphorical frames when producing news about this emerging information technology. These results also indicate that there is less exchange of information (in the ‘negotiation of meaning’) between journalists and other societal actors than is typically expected (Brüggeman, 2014; Cook, 1998; Gans, 1979).

Another dissimilarity in the use of metaphorical frames between journalists and experts is apparent in that journalists sometimes talked about cyberinfrastructure as a disruptive entity, while experts did not. Framing cyberinfrastructure as frightening appears to have originated from the journalists. According to the Extended Parallel Process Model (Witte, 1992), this can be problematic; when people perceive an emerging technology as a threat and if there is no solution to that threat, people might have defensive reactions, which implies that they may not adopt cyberinfrastructure. Conceptualizing the innovation this way is probably also not the best way to metaphorically frame cyberinfrastructure from a diffusion perspective.

Furthermore, framing emerging technologies as scary and dangerous is a regular occurrence in journalistic frames (Schwartz, 2018). Journalists often tend to exaggerate and sensationalize research findings (Jensen, 2008). This again indicates that, in creating frames, journalists primarily draw on their own metaphorical frames when constructing news about cyberinfrastructure. These metaphorical frames could possibly have negative effects on the public’s understanding of this information technology. More studies are needed to determine the effects of these metaphors on the audience’s perception of cyberinfrastructure, and the kind(s) of metaphorical frames that would lead to a more shared understanding of the form and function of cyberinfrastructure.

In terms of abstraction of the metaphorical frames, both journalists and experts used concrete metaphors. However, experts’ metaphors are more precise than those of journalists. This indicates that at the beginning of the frame-building process, it might be difficult for journalists to generate precise metaphors for topics that are complex and difficult to understand, while experts seem to have no problem with this. It is probably due to the fact that experts have more knowledge about cyberinfrastructure, allowing them to make more specific comparisons. Therefore, we expect that, when cyberinfrastructure becomes more widely adopted, and thus easier to understand, metaphorical frames of journalists will also become more precise. Future research should address this issue to provide insight into how metaphorical frames can change, which will in turn help create a better understanding of how the frame-building process evolves over time.

Our studies show that, for writing about emerging information technologies, journalists are not in the middle of the continuum of frame-sending and frame-setting (Brüggemann, 2014). Experts’ perspectives were not always reflected in the frames used by journalists, but journalists’ interpretations of cyberinfrastructure are likely the public’s first exposure to cyberinfrastructure. Therefore, journalists are closer to frame-setting than frame-sending on the continuum, as journalists often introduce metaphorical frames in public discourse (Kohring, 2005). Brüggemann (2014) argued that journalists would predominantly practice frame-setting when they were ‘acting in an environment that they perceive as providing consonant resonance for their own frames’ (p. 71). Because journalists might understand cyberinfrastructure in terms of the Internet, they may lean more towards frame-setting when conceptualizing cyberinfrastructure. The environment (context) of cyberinfrastructure provides consonant resonance for the Internet frames that are already being used in public discourse.

To conclude, journalists’ and experts’ metaphorical framing of cyberinfrastructure differed at the beginning of the frame-building process of cyberinfrastructure. Researchers interested in frame-building should thus take different relevant stakeholders into account, since they may differ considerably in their metaphor use. Journalists, to a large extent, employ their own frames in conceptualizing cyberinfrastructure rather than drawing on the frames used by experts, and therefore lean more towards frame-setting than the middle of the sending-versus-setting continuum. Future research could investigate the impact of these metaphorical frames on audience members. Finally, we must foster the interaction between these societal actors; by doing so, we can help create a shared understanding of the form and function of emerging information technologies.

## Notes

1. We used random.org to randomly select the articles.

2. Online Appendices A to G can be retrieved from the Open Science Framework (https://osf.io/q7nck/).

3. We compared the fixed 69 conceptual sub-domains of Study 1, with the 22 conceptual domains of Study 2 that were based on grounded theory (instead of the fixed 21 general conceptual domains of Study 1), because they were much more similar to each other in terms of their specificity.

## References

[bibr1-0963662520952542] Anthony L (2019) AntConc (Version 3.5.8, Computer Software). Tokyo, Japan: Waseda University Available at: https://www.laurenceanthony.net/software

[bibr2-0963662520952542] Atkins D (2003) Revolutionizing science and engineering through cyberinfrastructure: Report of the National Science Foundation blue-ribbon advisory panel on cyberinfrastructure. Available at: https://www.nsf.gov/cise/sci/reports/atkins.pdf (accessed 12 March 2018).

[bibr3-0963662520952542] Bauer A Bogner A (2020) Let’s (not) talk about synthetic biology: Framing an emerging technology in public and stakeholder dialogues. Public Understanding of Science 29: 492–507. 32122265 10.1177/0963662520907255 PMC7411530

[bibr4-0963662520952542] Brüggemann M (2014) Between frame setting and frame sending: How journalists contribute to news frames. Communication Theory 24(1): 61–82.

[bibr5-0963662520952542] Burgers C (2016) Conceptualizing change in communication through metaphor. Journal of Communication 66(2): 250–265.

[bibr6-0963662520952542] Burgers C Konijn EA Steen GJ (2016) Figurative framing: Shaping public discourse through metaphor, hyperbole, and irony. Communication Theory 26(4): 410–430.

[bibr7-0963662520952542] Campbell HA La Pastina AC (2010) How the iPhone became divine: New media, religion and the intertextual circulation of meaning. New Media & Society 12(7): 1191–1207.

[bibr8-0963662520952542] Charteris-Black J (2004) Corpus Approaches to Critical Metaphor Analysis. New York, NY: Palgrave Macmillan.

[bibr9-0963662520952542] Cook TE (1998) Governing with the News: The News Media as a Political Institution. Chicago, IL: University of Chicago Press.

[bibr10-0963662520952542] Cyberinfrastructure Council (2007) Cyberinfrastructure vision for 21st century discovery. Available at: https://www.nsf.gov/pubs/2007/nsf0728/nsf0728.pdf (accessed 16 March 2018).

[bibr11-0963662520952542] Entman RM (1993) Framing: Toward clarification of a fractured paradigm. Journal of Communication 43(4): 51–58.

[bibr12-0963662520952542] Foster I Kesselman C Tuecke S (2001) The anatomy of the grid: Enabling scalable virtual organizations. International Journal of High Performance Computing Applications 15(3): 200–222.

[bibr13-0963662520952542] Gans HJ (1979) Deciding What’s News. New York, NY: Pantheon.

[bibr14-0963662520952542] Hansen J Baumer EPS Richland L Tomlinson B (2011) Metaphor and creativity in learning science. In: Proceedings of the American educational researchers association annual conference (AERA), New Orleans, LA, 8–12 April.

[bibr15-0963662520952542] Hey T Trefethen AE (2005) Cyberinfrastructure for e-science. Science 308(5723): 817–821. 15879209 10.1126/science.1110410

[bibr16-0963662520952542] Iliev R Axelrod R (2017) The paradox of abstraction: Precision versus concreteness. Journal of Psycholinguistic Research 46(3): 715–729. 27878506 10.1007/s10936-016-9459-6

[bibr17-0963662520952542] Jaspal R Nerlich B (2014) Fracking in the UK press: Threat dynamics in an unfolding debate. Public Understanding of Science 23(3): 348–363. 23942831 10.1177/0963662513498835

[bibr18-0963662520952542] Jensen JD (2008) Scientific uncertainty in news coverage of cancer research: Effects of hedging on scientists’ and journalists’ credibility. Human Communication Research 34(3): 347–369.

[bibr19-0963662520952542] Joris W d’Haenens L Van Gorp B (2014) The Euro crisis in metaphors and frames: Focus on the press in the Low Countries. European Journal of Communication 29(5): 608–617.

[bibr20-0963662520952542] Kahn R Leiner BM Cerf VG Clark DD Kleinrock L Lynch DC, et al (1997) The evolution of the Internet as a global information system. The International Information & Library Review 29(2): 129–151.

[bibr21-0963662520952542] Kee KF (2015) Three critical matters in big data projects for e-science: Different user groups, the mutually constitutive perspective, and virtual organizational capacity. In: Proceedings of the 2015 IEEE international conference on Big Data, Santa Clara, CA, 29 October–1 November, pp. 2091–2097. New York: IEEE.

[bibr22-0963662520952542] Kee KF (2017) The 10 adoption drivers of open source software that enable e-research in data factories for open innovation. In: Matei S Julien N Goggins S (eds) Big Data Factories, Computational Sciences: Collaborative Approaches. New York, NY: Springer, pp. 51–65.

[bibr23-0963662520952542] Kee KF Cradduck L Blodgett B Olwan R (2011) Cyberinfrastructure inside out: Definitions and influences shaping its emergence, development, and implementation. In: Araya D Breindl Y Houghton T (eds) Nexus: New Intersections in Internet Research. New York, NY: Peter Lang, pp. 157–189.

[bibr24-0963662520952542] Kee KF Sleiman M Williams M Stewart D (2016) The 10 attributes that drive adoption and diffusion of computational tools in e-science. In: Navrátil P Dahan M Hart D Romanella A Sukhija N (eds) Proceedings of the 2016 XSEDE (Extreme Science & Engineering Discovery Environmental) conference New York, NY: ACM.

[bibr25-0963662520952542] Knudsen S (2005) Communicating novel and conventional scientific metaphors: A study of the development of the metaphor of genetic code. Public Understanding of Science 14(4): 373–392.

[bibr26-0963662520952542] Kohring M (2005) Wissenschaftsjournalismus: Forschungsüberblick und Theorieentwurf [Science journalism: Research overview and theory design]. Konstanz, Germany: UVK.

[bibr27-0963662520952542] Lacy L (2018) What a decentralized web means for digital advertising. Available at: https://www.adweek.com/programmatic/what-a-decentralized-web-means-for-digital-advertising/ (accessed 29 October 2018).

[bibr28-0963662520952542] Lakoff G Johnson M (2003 [1980]) Metaphors We Live by. Chicago, IL: The University of Chicago Press.

[bibr29-0963662520952542] Landis J Koch G (1977) The measurement of observer agreement for categorical data. Biometrics 33(1): 159–174. 843571

[bibr30-0963662520952542] Leydesdorff L Hellsten I (2005) Metaphors and diaphors in science communication: Mapping the case of stem cell research. Science Communication 27(1): 64–99.

[bibr31-0963662520952542] Luokkanen M Huttunen S Hildén M (2014) Geoengineering, news media and metaphors: Framing the controversial. Public Understanding of Science 23(8): 966–981. 23825283 10.1177/0963662513475966

[bibr32-0963662520952542] McCabe A (1988) Effect of different contexts on memory for metaphor. Metaphor and Symbol 3(2): 105–132.

[bibr33-0963662520952542] Markham AN (2003) Metaphors reflecting and shaping the reality of the Internet: Tool, place, way of being. In: Paper presented at the international association of Internet researchers, Toronto, ON, Canada, 16–19 October.

[bibr34-0963662520952542] Matlock T Castro SC Fleming M Gann TM Maglio PP (2014) Spatial metaphors of web use. Spatial Cognition & Computation 14(4): 306–320.

[bibr35-0963662520952542] Montreuil B (2011) Toward a physical Internet: Meeting the global logistics sustainability grand challenge. Logistics Research 3(2–3): 71–87.

[bibr36-0963662520952542] Palmquist RA (2001) Cognitive style and users’ metaphors for the web: An exploratory study. The Journal of Academic Librarianship 27(1): 24–32.

[bibr37-0963662520952542] Puschmann C Burgess J (2014) Big data, big questions: Metaphors of big data. International Journal of Communication 8: 1690–1709.

[bibr38-0963662520952542] Rogers EM (2003) Diffusion of Innovations, 5th edn. New York, NY: The Free Press.

[bibr39-0963662520952542] Scheufele DA (1999) Framing as a theory of media effects. Journal of Communication 49(1): 103–122.

[bibr40-0963662520952542] Schwartz O (2018) ‘The discourse is unhinged’: How the media gets AI alarmingly wrong. The Guardian, 25 7 Available at: https://www.theguardian.com/technology/2018/jul/25/ai-artificial-intelligence-social-media-bots-wrong (accessed 21 August 2018).

[bibr41-0963662520952542] Senyuva E Kaya H (2013) Metaphors for the Internet used by nursing students in Turkey: A qualitative research. Eurasian Journal of Educational Research 50: 87–106.

[bibr42-0963662520952542] Skorczynska H Deignan A (2006) Readership and purpose in the choice of economics metaphors. Metaphor and Symbol 21(2): 87–104.

[bibr43-0963662520952542] Sopory P Dillard JP (2002) The persuasive effects of metaphor: A meta-analysis. Human Communication Research 28(3): 382–419.

[bibr44-0963662520952542] Steen GJ Dorst AG Herrmann JB Kaal A Krennmayr T Pasma T (2010) A Method for Linguistic Metaphor Identification: From MIP to MIPVU. Amsterdam: Benjamins.

[bibr45-0963662520952542] Strauss A Corbin J (1990) Basics of Qualitative Research: Grounded Theory Procedures and Techniques. Thousand Oaks, CA: SAGE.

[bibr46-0963662520952542] Wear DN (1999) Challenges to interdisciplinary discourse. Ecosystems 2(4): 299–301.

[bibr47-0963662520952542] Williams-Whitney D Mio JS Whitney P (1992) Metaphor production in creative writing. Journal of Psycholinguistic Research 21(6): 497–509.

[bibr48-0963662520952542] Witte K (1992) Putting the fear back into fear appeals: The extended parallel process model. Communications Monographs 59(4): 329–349.

[bibr49-0963662520952542] Woods R Fernández A Coen S (2012) The use of religious metaphors by UK newspapers to describe and denigrate climate change. Public Understanding of Science 21(3): 323–339. 23045884 10.1177/0963662510385061

[bibr50-0963662520952542] Zhou J Chourasia A Vanderheiden G (2017) Interface adaptation to novice older adults’ mental models through concrete metaphors. International Journal of Human–Computer Interaction 33(7): 592–606.

